# The Educational Treatment of Idiots, Imbeciles, and Feeble-Minded Children

**Published:** 1894-07-21

**Authors:** G. E. Shuttleworth

**Affiliations:** late Medical Superintendent, Royal Albert Asylum, Lancaster


### The Educational Treatment of Idiots, Imbeciles, and Feeble-Minded Children.

By G. E. Shuttleworth, B.A., M.D., &c., late Medical Superintendent, Royal Albert Asylum, Lancaster.

### III.

Continuing our account (from issue of May 5th, 1894) of methods of training, we note the essentially practical character of the education of imbeciles. Without actually pursuing the system of "Dotheboys' Hall" (which Dickens has rendered famous), school work is continually brought into relation with the duties of daily life. In some institutions even "dressing lessons" form part of the school routine, with the object of inculcating that personal neatness which is conspicuous by its absence in the majority of cases. The practice of buttoning clothes, lacing boots, and tying strings is indeed very serviceable, apart from the practical applications, in improving the deficient muscular sense, and promoting the co-ordination of movements. With certain cases afflicted with spasmodic movements of the fingers (athetosis), the first efforts to master the mysteries of "buttoning up" are indeed painfully ludicrous. The vagrant fingers fly from the button as if electrified, and it requires a strong effort of the will to bring them back to the prescribed spot. But in this, as in other things, practice makes perfect, and the perseverance of the pupil is at last rewarded by an aptitude which, in some cases is extraordinary. Difficult macrame work, in which an intricate pattern is correctly followed, is often accomplished by children who could not at first tie a knot; and drawings of considerable merit are made by pupils unable, before training, even to grasp a pencil. It is indeed remarkable that patients of the "athetotic" type frequently display a considerable graphic ability.

Many of the occupations of the Kindergarten are utilised for finger exercises and manual training, though the system of Fröbel, depending mainly upon the spontaneous activity of child-life, which is lacking with idiots, is not applicable in its entirety. Exercises of imitation, indeed, are requisite for this class; and on this account, as well as others, great care is necessary in the selection of teachers and the classification of pupils. To be a successful teacher of imbeciles, it is necessary to be able to adapt the principles of Pestalozzi and Fröbel to the special conditions of the individual pupil; and an aptitude for industrial training is also essential. It has been well remarked that, as a rule, this class "learn more with their hands than their heads," and the experience of training institutions for imbeciles had anticipated by many years the arrangements of which we have heard so much of late in connection with technical education in elementary schools.

One of the practical lessons of the idiot school is, perhaps, worthy of special mention, as, in our opinion, something similar might advantageously be introduced into the ordinary school. It is an arrangement for impressing upon the children's minds, in concrete form, the significance of weights and measures and the value of money, and is called the "Shop-lesson." On one side of the class-room, facing the children's "gallery," stands a cabinet, fitted with canisters and drawers, containing tea, sugar, raisins, and other groceries, and, in addition, on the girls' side, tapes, ribbons, &c. In front of the cabinet is a counter, on which are scales and weights, measures, various coins of the realm, and also wrapping materials. The teacher selects a "shopkeeper," and then calls upon the pupils in turn to make purchases. The problem of the "shopkeeper" is to select, weigh or measure, and wrap up the article in the quantity asked for; that of the "purchaser" to reckon up and pay correctly for the article bought, taking care that he receives proper change. The lesson is, indeed, only an expansion of the old nursery game of "shop"; but, under a judicious teacher, it may be made the vehicle of valuable manual and mental training, and is, moreover, of direct practical utility in the routine of everyday life.

We have previously referred to some of the ingenious arrangements devised by Séguin and others for the cultivation of the tactile sense. The practice of bead-threading is also very serviceable for this purpose, offering gradations of difficulty according to the size of the beads used. In addition to being a finger exercise, it becomes, when the beads are threaded in series of number and colour (*e.g.*, two black and two white, three red and two blue, and so on), a lesson in counting and in colour selection, and at the same time the result in variegated necklaces fascinates the feeble mind to fresh efforts. "Little things please little minds," and observance of this maxim may make all the difference between rendering school work attractive or repulsive to the imbecile.

Calculation is, of course, the "crux" of the mentally feeble pupil. He may perhaps grasp the concrete, but the abstract will always be imperfectly appreciated. Illustrations of numbers by pictures and objects, and objective demonstrations of arithmetical operations are consequently indispensable. The bead frame (*abacus*) and other ingenious arrangements for ocular calculations are of special value; and the game of dominoes is serviceable in pleasantly helping to an understanding of numbers.

In comparing the school routine of an imbecile and of a normal child, we must not forget that the latter learns much instinctively without conscious effort, while the child isolated by mental infirmity from his fellows has to be taught methodically what the other "picks up." Consequently, such matters as the names and uses of the parts of the body, and of various articles of clothing, distinguishing colours, and telling time by the clock, fall within the scope of the special teacher; and it should not therefore excite surprise that the progress of a pupil thus handicapped mu.t of necessity be slow.

